# Ultrasound‐Indicated Versus Physical Examination‐Indicated Cerclage in Twin Pregnancies Between 24 and 27 + 6 Weeks: An Exploratory Observational Analysis of Pregnancy and Neonatal Outcomes

**DOI:** 10.1155/jp/2647297

**Published:** 2026-10-14

**Authors:** Peng Zhao, Xiaomin Wang, Lidan Zhang, Lifeng Zhang, Mengyang Hu, Jawad Safdar, Kai Zhang, Maksim Beluga, Ivan Kurlovich

**Affiliations:** ^1^ Assisted Reproduction Unit Department of Obstetrics and Gynecology Sir Run Run Shaw Hospital School of Medicine Zhejiang University Hangzhou China zju.edu.cn; ^2^ Zhejiang Provincial Clinical Research Center for Reproductive Health and Disease Hangzhou China; ^3^ Zhejiang Key Laboratory of Precise Protection and Promotion of Fertility Hangzhou China; ^4^ Department of Obstetrics Women′s Hospital Zhejiang University School of Medicine Hangzhou China zju.edu.cn; ^5^ Department of Oral and Maxillofacial Surgery Dow Dental College Dow University of Health Sciences Karachi Pakistan duhs.edu.pk; ^6^ Department of Ultrasound in Medicine the Fourth Affiliated Hospital of School of Medicine and International School of Medicine International Institutes of Medicine Zhejiang University Yiwu China zju.edu.cn; ^7^ Key Laboratory of Surface and Interface Science of Polymer Materials of Zhejiang Province School of Chemistry and Chemical Engineering Zhejiang Sci-Tech University Hangzhou China zstu.edu.cn; ^8^ State Institution Republican Scientific and Practical Center “Mother and Child” of the Ministry of Health of the Republic of Belarus Minsk Belarus

**Keywords:** cervical cerclage, physical examination-indicated cerclage, preterm birth, short cervix, twin pregnancies, ultrasound-indicated cerclage

## Abstract

**Background:**

Recent advancements in assisted reproductive technology and delayed parenthood have led to a surge in twin pregnancies, with a high incidence of preterm birth (PTB) due to cervical insufficiency. Current guidelines recommend cervical cerclage for twin pregnancies before 24 weeks; however, data on cerclage efficacy beyond 24 weeks is scarce.

**Objective:**

This exploratory, hypothesis‐generating study is aimed at examining the associations of cervical cerclage on pregnancy duration and neonatal outcomes in twin pregnancies with a short cervix between 24 and 27 + 6 weeks.

**Methods:**

This retrospective cohort study, conducted between July 1991 and July 2023 at Women′s Hospital, Zhejiang University School of Medicine, involved twin pregnancies with a short cervix (≤ 25 mm) during the gestational period ranging from 24 weeks to 27 + 6 weeks. Skilled obstetricians performed cervical cerclage. Data on maternal and neonatal outcomes were collected and analyzed using statistical tests, with statistical significance set at *p* < 0.05.

**Results:**

A total of 73 twin pregnancies were analyzed. Participants were categorized into ultrasound‐indicated cerclage group (*n* = 22), physical examination‐indicated cerclage group (*n* = 33), and control group (*n* = 18). The ultrasound‐indicated cerclage group showed significantly longer presentation‐to‐birth intervals (60.77 ± 21.64 days, *p* = 0.007) and higher birth weights (larger twin: 2273.33 ± 566.09 g, *p* = 0.026; smaller twin: 2086.90 ± 526.58 g, *p* = 0.029) compared with the control group. No significant differences were found in gestational age at delivery, Apgar scores, cesarean delivery rates, maternal morbidity, and neonatal morbidity among both groups.

**Conclusions:**

Ultrasound‐indicated cerclage in twin pregnancies at 24–27 + 6 weeks was observed to prolong presentation‐to‐birth interval and increase birth weight, whereas physical examination‐indicated cerclage showed no statistically significant association with the measured outcomes. A randomized controlled clinical trial is needed to validate these findings.

## 1. Introduction

In recent years, the advancement of assisted reproductive technology coupled with the rising trend of delayed childbearing has led to a notable increase in the incidence of twin pregnancies [1]. The prevalence of twin pregnancy has been reported to be 3.12% in the United States and 3.69% in China [2, 3]. Preterm birth (PTB), which stands as the foremost complication in twin pregnancies, boosts an alarmingly high incidence of 61.27% [2]. A significant factor contributing to PTB is cervical insufficiency, which is seven times more prevalent in twin pregnancies compared with singleton pregnancies and is most commonly observed in primiparous women, manifested as a shortened cervix [4].

Although cervical cerclage is commonly accepted in singleton pregnancies with historical indications, physical examination findings or ultrasonographic criteria [5], its application in twin pregnancies has elicited considerable debate and controversy. One of the major debates centered on the question of whether cervical cerclage should be performed in twin pregnancies beyond 24 weeks of gestation. According to clinical guidelines, cerclage is recommended for twin pregnancies demonstrating cervical dilation of ≥ 10 mm, or for those with a cervical length of < 15 mm, before the completion of the 24th week of gestation [5–7]. Moreover, for twins between 24 and 30 weeks of gestation, cervical shortening was also found to be associated with an increased risk of PTB [8]. Nevertheless, these guidelines do not provide clear direction for cases involving twin pregnancies beyond the 24th week of gestation. Recent studies have undertaken a more thorough evaluation on the efficacy of cerclage in twin pregnancies before 24 weeks of gestation [9, 10], whereas the available studies exploring its benefits after 24 weeks are exceedingly scarce, leaving a great gap in attention for these patients.

Therefore, we performed this exploratory, hypothesis‐generating study with the objective to describe pregnancy duration, as well as maternal and neonatal outcomes following cervical cerclage, in twin pregnancies with short cervix between 24 and 27 + 6 weeks of gestation.

## 2. Methods

This retrospective cohort study was conducted at Women′s Hospital, Zhejiang University School of Medicine between July 1991 and July 2023. The analysis was designed as exploratory and hypothesis‐generating, with the aim of informing future prospective research rather than establishing clinical recommendations for cerclage in twin pregnancies beyond 24 weeks. The participants were all twin pregnancies with uncomplicated medical conditions but exhibited short cervix (cervical length ≤ 25 mm under transvaginal ultrasound) during the gestational period ranging from 24 weeks to 27 + 6 weeks. We chose short cervix as the inclusion criterion rather than a diagnosis of cervical insufficiency, as mentioned in the Introduction, because the majority of cervical incompetence cases in twin pregnancies are observed in primiparae [4].

The exclusion criteria were 1) clinical chorioamnionitis, 2) preterm premature rupture of membranes (PPROM), 3) established labor, 4) cervical dilation ≥ 30 mm [11], 5) complicated monochorionic twins (twin‐to‐twin transfusion syndrome, selective intrauterine growth restriction; twin reversed arterial perfusion sequence; twin anemia‐polycythemia sequence, etc.), 6) complicated dichorionic twins; 7) triplets and higher‐order multiples. All participants had been sufficiently informed about the risks and potential benefits of cervical cerclage and expectant management. The ultimate decision regarding treatment was made by patients after consultation and discussion with their attending physician.

Based on their individual treatment strategy, the participants were subsequently categorized into three distinct groups: the ultrasound‐indicated cerclage group (cervical length ≤ 25 mm with no cervical dilation), the physical examination‐indicated cerclage group (cervical dilation ≥ 10 mm but < 30 mm) and the control group (expectant management group), as illustrated in Figure 1. Cervical cerclage procedures were performed by skilled chief obstetricians utilizing either the McDonald or Shirodkar technique with precision and expertise. Repeated ultrasound monitoring of the cervical length was routinely provided. Prophylactic antibiotics and tocolytics were administered when deemed necessary. The cervical cerclage was typically removed at 36–37 weeks of gestation, in the event of labor onset, PPROM or the development of chorioamnionitis. Participants in control group received a range of conservative treatments, including bed rest, oral progesterone therapy, tocolytic agents, regular surveillance with ultrasound, lifestyle changes.

**Figure 1:**
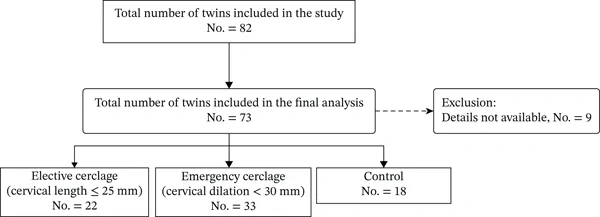
Flow diagram.

The following data were obtained: maternal age, gravidity, parity, chorionicity, gestational age (GA) at presentation, GA at presentation, presentation to birth interval, mode of delivery, birth weight, Apgar scores, caesarean delivery rate, neonatal morbidity, and maternal morbidity. The primary outcomes of the current study were presentation to birth interval and birth weight. The second outcomes included Apgar scores, neonatal morbidity, and maternal morbidity.

Continuous variables are presented as means and standard deviations; categorical variables are presented as percentages and counts. Data were compared by the chi‐square test for categorical variables and independent *t*‐test for continuous variables. To identify factors independently associated with the presentation to birth interval, larger twin birth weight, and smaller twin birth weight. The models included the following five prespecified covariates: progesterone use (0, not used; 1, used; reference, 0), ultrasound‐indicated cerclage (0, no; 1, yes; reference, 0), maternal comorbidity (0, no; 1, yes; reference, 0), chorionicity (0, monochorionic diamniotic; 1, dichorionic diamniotic; reference, 0), and GA at presentation (0, 24–25 weeks; 1, 26–27 weeks; reference, 0). Variable selection was based on a priori clinical relevance rather than through data‐driven stepwise procedures, to minimize overfitting given the small sample size. Model fit was assessed using the scaled deviance and scaled Pearson chi‐square statistics divided by their residual degrees of freedom; values close to 1.0 indicate adequate fit. The omnibus likelihood ratio test was used to evaluate the overall significance of the fitted model. Adjusted regression coefficients (B) were reported with 95% Wald confidence intervals and *p*‐values from Wald chi‐square tests. A *p* − value < 0.05 was considered statistically significant. All statistical analyses were performed using IBM SPSS (Version 25.0, IBM Corp., Armonk, New York, United States) for Windows.

## 3. Results

### 3.1. Participants Selection

A total of 82 twins were enrolled according to the criteria, of whom 9 were excluded for incomplete data. Finally, 73 women with twin pregnancies were divided into ultrasound‐indicated cerclage group (*n* = 22), physical examination‐indicated cerclage group (*n* = 33) and control group (*n* = 18). Details were presented in Figure 1.

### 3.2. Baseline Clinical Characteristics

In comparison with the control group, no statistically significant differences were noted pertaining to maternal age, gravidity, parity, PTB, midterm painless abortion, and dichorionic twins in the ultrasound‐indicated cerclage group (maternal age: 30.45 ± 4.50 vs. 30.06 ± 4.81, *p* = 0.790; gravidity: 1.73 ± 1.08 vs. 2.22 ± 1.35, *p* = 0.205; parity: 0.09 ± 0.29 vs. 0.39 ± 0.61, *p* = 0.069; history of spontaneous PTB: 0 vs. 12.50%, *p* = 0.196; history of midterm painless abortion: 0 vs. 0, dichorionic diamniotic twins: 90.91% vs. 94.44%, *p* = 1.000) and the physical examination‐indicated group (maternal age: 29.33 ± 3.22 vs. 30.06 ± 4.81, *p* = 0.524; gravidity: 1.82 ± 1.31 vs. 2.22 ± 1.35, *p* = 0.303; parity: 0.12 ± 0.33 vs. 0.39 ± 0.61, *p* = 0.097; history of spontaneous PTB: 0 vs. 11.11%, *p* = 0.120; history of midterm painless abortion: 6.06% vs. 0, *p* = 0.534; dichorionic diamniotic twins: 90.91% vs. 94.44%, *p* = 1.000). Moreover, both cerclage groups had significantly lower mean GAs at presentation (ultrasound‐indicated: 24.50 ± 0.74 weeks, *p* < 0.001; physical examination‐indicated: 24.73 ± 0.91 weeks, *p* = 0.001) compared with controls (25.72 ± 1.13 weeks), with higher proportions of cases at 24–25 weeks (ultrasound‐indicated: 86.36% vs. 44.44%, *p* = 0.005; physical examination‐indicated: 75.76% vs. 44.44%) and fewer at 26–27 weeks (ultrasound‐indicated: 13.64% vs. 55.56%; physical examination‐indicated: 24.24% vs. 55.56%, *p* = 0.025) than the control group. Details were presented in Tables 1 and 2.

**Table 1:** Comparison between the ultrasound‐indicated cerclage group and the control group.

Measurements	Ultrasound‐indicated cerclage	Control	*p*
	*N* = 22	*N* = 18	
Clinical characteristics			
Maternal age	30.45 ± 4.50	30.06 ± 4.81	0.790
Gravidity	1.73 ± 1.08	2.22 ± 1.35	0.205
< 1	14	7	0.119
≥ 1	8	11	
Parity	0.09 ± 0.29	0.39 ± 0.61	0.069
< 1	20	12	0.110
≥ 1	2	6	
History of midterm painless abortion	0 (0/22)	0 (0/18)	NA
History of spontaneous preterm birth	0 (0/22)	11.11% (2/18)	0.196
Chorionicity			
Dichorionic diamniotic twins	90.91% (20/22)	94.44% (17/18)	1.000
Monochorionic diamniotic twins	9.09% (2/22)	5.56% (1/18)	
Gestational age at presentation (weeks)	24.50 ± 0.74	25.72 ± 1.13	< 0.001
24–25	86.36% (19/22)	44.44% (8/18)	0.005
26–27	13.64% (3/22)	55.56% (10/18)	
Pregnancy outcomes			
Gestational age at delivery (weeks)	33.23 ± 2.96	31.50 ± 3.67	0.107
Presentation to birth interval (days)	60.77 ± 21.64	40.50 ± 23.11	0.007
Birth weight (grams)			
Larger	2273.33 ± 566.09	1836.11 ± 610.03	0.026
Smaller	2086.90 ± 526.58	1680.83 ± 593.27	0.029
Apgar score 1 min			
Larger	9.27 ± 1.12	8.00 ± 3.16	0.119
Smaller	9.82 ± 0.40	8.72 ± 3.20	0.166
Apgar score 5 min			
Larger	9.82 ± 0.40	8.72 ± 3.20	0.083
Smaller	9.77 ± 0.75	8.56 ± 3.17	0.128
Caesarean delivery	94.45% (21/22)	83.33% (15/18)	0.310
Neonatal morbidity	71.43% (15/21)^a^	72.22% (13/18)	0.956
Maternal morbidity	81.82% (18/22)	94.44% (17/18)	0.355

^a^Indicates that data for one participant were unobtainable.

**Table 2:** Comparison between the physical examination‐indicated cerclage group and the control group.

Measurements	Physical examination‐indicated cerclage	Control	*p*
	*N* = 33	*N* = 18	
Clinical characteristics			
Maternal age	29.33 ± 3.22	30.06 ± 4.81	0.524
Gravidity	1.82 ± 1.31	2.22 ± 1.35	0.303
< 1	20	7	0.138
≥ 1	13	11	
Parity	0.12 ± 0.33	0.39 ± 0.61	0.097
< 1	29	12	0.068
≥ 1	4	6	
History of midterm painless abortion	6.06% (2/33)	0 (0/18)	0.534
History of spontaneous preterm birth	0 (0/33)	11.11% (2/18)	0.120
Chorionicity			
Dichorionic diamniotic twins	90.91% (30/33)	94.44% (17/18)	1.000
Monochorionic diamniotic twins	9.09% (3/33)	5.56% (1/18)	
Gestational age at presentation (weeks)	24.73 ± 0.91	25.72 ± 1.13	0.001
24–25	75.76% (25/33)	44.44% (8/18)	0.025
26–27	24.24% (8/33)	55.56 (10/18)	
Pregnancy outcomes			
Gestational age at delivery (weeks)	30.52 ± 3.31	31.50 ± 3.67	0.350
Presentation to birth interval (days)	41.24 ± 22.79	40.50 ± 23.11	0.912
Birth weight (grams)			
Larger	1617.58 ± 541.20	1836.11 ± 610.03	0.213
Smaller	1436.52 ± 440.00	1680.83 ± 593.27	0.101
Apgar score 1 min			
Larger	8.18 ± 2.65	8.00 ± 3.16	0.828
Smaller	8.21 ± 2.41	7.50 ± 3.22	0.376
Apgar score 5 min			
Larger	9.09 ± 2.01	8.72 ± 3.20	0.615
Smaller	9.18 ± 2.05	8.55 ± 3.17	0.396
Caesarean delivery	66.67% (22/33)	83.33% (15/18)	0.202
Neonatal morbidity	84.85% (28/33)	72.22% (13/18)	0.278
Maternal morbidity	90.63% (29/32)^a^	94.44% (17/18)	1.000

^a^Indicates that data for one participant were unobtainable.

### 3.3. Pregnancy Outcomes

For the ultrasound‐indicated cerclage group, in comparison with the control group, presentation to birth interval (days) was significantly increased (60.77 ± 21.64 vs. 40.50 ± 23.11, *p* = 0.007). Similarly, birth weight (grams) was notably elevated (for the larger: 2273.33 ± 566.09 vs. 1836.11 ± 610.03, *p* = 0.026; for the smaller: 2086.90 ± 526.58 vs. 1680.83 ± 593.27, *p* = 0.029). No significant differences were noted in GA at delivery, Apgar scores, caesarean delivery rate, neonatal morbidity, and maternal morbidity. In our study, neonatal morbidity is defined as a wide range of severe health complications occurring within the first 28 days of an infant′s life [12]. Maternal morbidity refers to any health condition attributed to or aggravated by pregnancy and childbirth that negatively impacts a woman′s well‐being [13]. Details were presented in Table 1.

For the physical examination‐indicated cerclage group, in comparison with the control, a slight increase was noted in presentation to birth interval (41.24 ± 22.79 vs. 40.50 ± 23.11, *p* = 0.912), Apgar score 1 min (for the larger:8.18 ± 2.65 vs. 8.00 ± 3.16, *p* = 0.828; for the smaller: 8.21 ± 2.41 vs. 7.50 ± 3.22, *p* = 0.376), Apgar score 5 min (for the larger: 9.09 ± 2.01 vs. 8.72 ± 3.20, *p* = 0.615; for the smaller: 9.18 ± 2.05 vs. 8.55 ± 3.17, *p* = 0.396), and lower birth weight (for the larger: 1617.58 ± 541.20 vs. 1836.11 ± 610.03, *p* = 0.213; for the smaller: 1436.52 ± 440.00 vs. 1680.83 ± 593.27, *p* = 0.101), although no significant differences were detected. No significant differences were noted in GA at delivery, caesarean delivery rate, neonatal morbidity, and maternal morbidity. Details were presented in Table 2.

To identify factors independently associated with the presentation to birth interval and birth weight, multivariable generalized linear regression analysis was performed. Model fit was adequate, with adjusted deviance/df and adjusted Pearson chi‐square/df approximately 1.0 (presentation to birth interval, 1.176; larger twin birth weight, 1.182; smaller twin birth weight, 1.182). The omnibus likelihood ratio test was significant in all three models (presentation to birth interval: *χ* ^2^ = 19.734, *p* = 0.001; larger twin birth weight: *χ* ^2^ = 18.787, *p* = 0.002; smaller twin birth weight: *χ* ^2^ = 22.059, *p* = 0.001). After adjusting for potential confounders, ultrasound‐indicated cerclage was found to be a significant predictor of extended presentation to birth interval and increased birth weight. Specifically, patients who received ultrasound‐indicated cerclage experienced a mean increase of 19.23 days (*p* = 0.007) in the presentation to birth interval and a mean increase of 598.71 g for larger twin and 544.21 g for smaller twin in birth weight (both *p* = 0.001) compared with the control group. Progesterone use exhibited a mean increase of 18.89 days (*p* = 0.008) in the presentation to birth interval and a mean increase of 412.10 g for larger twin and 476.14 g for smaller twin in birth weight (*p* = 0.032 and *p* = 0.006, respectively). Dichorionic diamniotic did not significantly prolong the pregnancy interval, but it was associated with a mean birth‐weight increase of 972.96 g for the larger twin and 963.69 g for the smaller twin (*p* = 0.010 and *p* = 0.005, respectively). In contrast, GA at presentation and maternal morbidity were not significantly associated with either outcome. Details were presented in Tables 3 and 4.

**Table 3:** Multivariable generalized linear regression analysis for factors associated with presentation‐to‐birth interval (days).

Variable	*β* (SE)	95% Wald confidence interval (lower limit, upper limit)	*W* *a* *l* *d* *χ*2	*p*
Intercept	5.307 (19.343)	−32.604, 43.218	0.075	0.784
Ultrasound‐indicated cerclage	19.230 (7.175)	5.167, 33.292	7.183	0.007
Progesterone use	18.893 (7.134)	4.910, 32.876	7.013	0.008
Chorionicity	27.631 (14.586)	−0.957, 56.218	3.589	0.058
Gestational age at presentation	−5.182 (7.428)	−19.740, 9.376	0.487	0.485
Maternal morbidity	−3.393 (9.202)	−21.427, 14.642	0.136	0.712

**Table 4:** Multivariable generalized linear regression analysis for factors associated with birth weight (g).

Variable	*β* (SE)	95% Wald confidence interval (lower limit, upper limit)	*W* *a* *l* *d* *χ*2	*p*
Larger twin				
Intercept	410.773 (497.681)	−564.663, 1386.210	0.681	0.409
Ultrasound‐indicated cerclage	598.712 (187.858)	230.518, 966.906	10.157	0.001
Progesterone use	412.101 (192.152)	35.491, 788.711	4.600	0.032
Chorionicity	972.958 (377.798)	232.487, 1713.428	6.632	0.010
Gestational age at presentation	248.091 (190.743)	−125.758, 621.940	1.692	0.193
Maternal morbidity	17.918 (237.372)	−447.323, 483.160	0.006	0.940
Smaller twin				
Intercept	241.562 (452.716)	−645.745, 1128.870	0.285	0.594
Ultrasound‐indicated cerclage	544.212 (170.885)	209.284, 879.141	10.142	0.001
Progesterone use	476.138 (174.791)	133.554, 818.721	7.420	0.006
Chorionicity	963.690 (343.664)	290.120, 1637.260	7.863	0.005
Gestational age at presentation	207.340 (173.510)	−132.732, 547.413	1.428	0.232
Maternal morbidity	17.486 (215.926)	−405.721, 440.694	0.007	0.935

## 4. Discussion

### 4.1. Main Findings

In this exploratory study, the principal observations were: 1) After adjustment, a significantly longer interval from presentation to birth and higher twin birth weight were observed following ultrasound‐indicated cerclage; however, no significant difference was noted in GA at delivery. 2) No comparable prolongation of pregnancy duration was noted following physical examination‐indicated cerclage.

### 4.2. Comparison With Prior Work

It was a well‐established fact that twin pregnancies carried a heightened risk of PTB compared with singleton pregnancies, particularly in those individuals with a shortened cervix [14]. Cervical cerclage has proven effective in improving the outcomes of twin pregnancies with a short cervix before 24 weeks [15]. However, investigations into this procedure beyond the 24th week remain limited. A study conducted by Yao et al. [16] involved 320 twin pregnancies with a short cervix (cervical length ≤ 25 mm) occurring between 16 and 28 weeks of gestation. Among these participants, 118 women were beyond the 24th week of pregnancy, with 44 women assigned to the cerclage group and 74 women to the control group. Consequently, there were no statistically significant differences in terms of perinatal mortality (4.55% vs. 1.35%; *p* = 0.132), low birth weight (59.09% vs. 70.27%; *p* = 0.089), very low birth weight (15.91% vs. 9.46%; *p* = 0.150), NICU admission (35.71% vs. 46.58%; *p* = 0.128), median birth weight (2405 g vs. 2325 g; *p* = 0.973), and the GA at delivery (34.73 ± 3.08 weeks vs. 34.91 ± 2.43 weeks; *p* = 0.722). Another 12‐year retrospective cohort study performed by Tan et al. arrived at the same conclusion [17]. The authors enrolled twin pregnancies with asymptomatic cervical dilation, visible membranes by speculum examination or a short cervical length of ≤ 25 mm as determined by transvaginal ultrasound. A total of 58 women were enrolled with 37 being less than 24 weeks of gestation and 21 women between 24 and 28 weeks of gestation. In the subgroup of women between 24 and 28 weeks of gestation, the cerclage group exhibited no significant differences in GA at delivery, interval from presentation to delivery and birth weight (*p* > 0.05). The results of the aforementioned two studies indicated that cervical cerclage did not offer any advantages over the control group, which contradicted our findings. We postulate that a significant confounding factor in their studies was the lack of stratification of participants based on cervical dilation. In contrast, our study did stratify participants into two groups: an ultrasound‐indicated cerclage group (with a nondilated cervix) and a physical examination‐indicated cerclage group (with a dilated cervix). Notably, the ultrasound‐indicated cerclage group demonstrated significant advantages over the physical examination‐indicated cerclage group.

Our study finds support in a previous study conducted by Qiu et al., which employed a stratification method similar to ours. Their study included 40 twin pregnancies, with a GA ranging from 24 to 26 weeks, and was characterized by a shortened cervix identified via transvaginal ultrasound examination. Importantly, none of the participants had a dilated cervix. They concluded that the ultrasound‐indicated cerclage group, which was comparable to the ultrasound‐indicated cerclage group in our study, had a significantly higher GA at delivery (34.53 ± 1.95 vs. 31.24 ± 3.00 weeks, *p* < 0.001), a longer pregnancy latency (70.58 ± 16.26 vs. 43.43 ± 24.68 days, *p* < 0.001) and a lower incidence of spontaneous PTB (*p* < 0.05) [18].

It is noteworthy to mention that both ultrasound‐indicated and physical examination‐indicated cerclage groups exhibited significantly lower mean GAs at presentation compared with controls. Further analysis revealed a higher proportion of cases presenting at 24–25 weeks and a correspondingly lower proportion at 26–27 weeks across both groups. This distribution likely reflects how GA shapes clinical decision‐making. As fetal viability improves with advancing pregnancy, management of feasible pregnancies appropriately shifts toward conservative, noninvasive approaches over surgical interventions, thereby avoiding surgical risks while maintaining favorable perinatal outcomes.

### 4.3. Clinical Implications

Previous studies have reported that among neonates delivered at 24–27 weeks of gestation, mortality and major morbidity rates were alarmingly high, at 14.67% and 49.47%, respectively, with only 1.77% surviving without major complications, whereas beyond 28 weeks of gestation, neonatal survival rates rise dramatically to 98% [19]. Reaching the critical threshold of 28 weeks of gestation is therefore critical for neonatal survival. In the present cohort, ultrasound‐indicated cervical cerclage prolonged the presentation‐to‐birth interval, with a mean increase of 60.77 ± 21.64 days from procedure to delivery. Our data suggest that for twin pregnancies managed with ultrasound‐indicated cerclage at 24 weeks of gestation, there may be a trend toward delivery after the critical 28‐week threshold, despite the lack of a significant difference in gestational age at delivery.

### 4.4. Limitations

The main limitations of our study lie in its retrospective nature and the relatively limited number of samples analyzed. First, this retrospective study may have introduced inherent biases, as certain data were unavailable for some participants. Second, because treatment allocation was nonrandomized and based on shared decision‐making between patients and physicians, differences in GA at presentation were observed between groups, suggesting the possibility of indication bias. Third, the limited sample size may have compromised statistical power and the generalizability of our findings. Fourth, despite adjusting for multiple covariates, the absence of key disease severity markers may result in residual confounding; thus, our findings should be regarded as exploratory and hypothesis‐generating rather than definitive, and causal interpretations should be made with caution regarding unmeasured factors.

## 5. Conclusions

Among twin pregnancies with a cervical length < 25 mm at 24–27 + 6 weeks of gestation, ultrasound‐indicated cerclage was observed to be associated with longer presentation‐to‐birth interval and higher birth weight, however, no significant difference in GA at delivery or measured neonatal outcomes was observed. Physical examination‐indicated cerclage showed no significant association with the outcomes assessed. A large‐scale, multicenter randomized controlled trial would be essential to validate and further consolidate our results.

**Nomenclature**
df: degrees of freedom
GA: gestational age
NICU: neonatal intensive care unit
PPROM: preterm premature rupture of membranes
PTB: preterm birth
vs: versus

## Author Contributions

Xiaomin Wang: conceptualization, data curation, investigation, writing—original draft. Lifeng Zhang, Mengyang Hu, Jawad Safdar, Maksim Beluga, and Ivan Kurlovich: investigation, data curation, writing—review and editing. Lidan Zhang and Kai Zhang: formal analysis, validation, and visualization. Peng Zhao: conceptualization, methodology, supervision, writing—review and editing.

## Funding

No funding was received for this manuscript.

## Disclosure

All authors critically reviewed and approved the final manuscript.

## Ethics Statement

The current study was granted approval by the Ethics Committee of the Women′s Hospital, School of Medicine, Zhejiang University (IRB‐20240227‐R), and the requirement for written informed consent was waived. All procedures were conducted in accordance with the Declaration of Helsinki to protect personal data.

## Conflicts of Interest

The authors declare no conflicts of interest.

## Data Availability

The data that support the findings of this study are available from the corresponding author upon reasonable request.
